# Inherent characteristics of metachronous metastatic renal cell carcinoma in the era of targeted agents

**DOI:** 10.18632/oncotarget.20230

**Published:** 2017-08-12

**Authors:** Jang Hee Han, Seung Hwan Lee, Won Sik Ham, Woong Kyu Han, Koon Ho Rha, Young Deuk Choi, Sung Joon Hong, Young Eun Yoon

**Affiliations:** ^1^ Department of Urology, Urological Science Institute, Yonsei University College of Medicine, Seoul, Korea; ^2^ Department of Urology, Hanyang University College of Medicine, Seoul, Korea

**Keywords:** renal cell carcinoma, targeted therapy, metastasis, prognosis, survival

## Abstract

**Background:**

To assess the prognostic and predictive factors of time to treatment failure (TTF) and overall survival (OS), respectively, in patients with metachronous metastatic renal cell carcinoma (mRCC) who were treated with targeted agents.

**Materials and Methods:**

We retrospectively reviewed metachronous mRCC patients, defined as individuals diagnosed with metastatic disease >3 months after initial nephrectomy, treated at an institute since 2005. Cox proportional hazard regression analysis was performed to discover the most determinant variables associated with TTF and OS.

**Results:**

Sarcomatoid features, absence of metastasectomy, multiple site metastasis, time to metastasis <1.5 year, and increased corrected calcium were independent prognostic factors of OS. The low risk group (0–1 risk factors) did not reach the median OS, whereas the OS for the intermediate (2 risk factors) and high risk groups (3–5 risk factors) were 58.6 and 23.6 months, respectively (p<0.001). When a death event was considered the dependent factor, the area under the receiver operating characteristic curve was significantly higher than in the existing International mRCC Database Consortium (IMDC; p=0.010) and Memorial Sloan Kettering Cancer Center (MSKCC; p=0.010) risk criteria models.

**Conclusion:**

Initial tumor size or T stage did not affect TTF or OS. Patients who could not undergo metastasectomy and rapidly developed multiple metastases with higher corrected calcium and initial tumors with sarcomatoid features were less likely to benefit from targeted therapy; thus, the new agents under development or clinical trials could be more helpful than the use of standard targeted agents.

## INTRODUCTION

The surgical resection of localized renal cell carcinoma (RCC) results in a 5-year survival of approximately 90% [1]. However, widespread metastatic RCC (mRCC) develops in 30% to 40% of patients after the initial resection [2]. Further, there is a 7% chance of metachronous metastatic disease up to 5 years after nephrectomy and a 16% chance at 10 years [3]. Due to the high incidence of metastasis, the management of mRCC has been revolutionized by therapeutic targeting of molecular pathways, which results in improved tumor response and prolonged survival [1].

Although the use of targeted agents has dramatically improved the prognosis of mRCC patients, complete remission rates remain poor and resistance to targeted therapies is high [4-6]. Consequently, several other treatment modalities including surgical resection (metastasectomy), radiotherapy, and classical immune therapy are still used to extend overall survival (OS) rates [7, 8]. Furthermore, we are currently awaiting the approval and availability of the next generation of immune checkpoint inhibitors, which are currently under clinical trials [9-11]. Hence, defining poor responders or those with increased resistance to targeted agents will significantly impact treatment planning outcomes.

Metastatic cancer is generally divided into synchronous and metachronous categories by the period between primary cancer treatment and the occurrence of metastasis, respectively. In the era of immune-based therapies, these two mRCC groups were investigated extensively and compared for inherently different characteristics, which revealed better survival rates in the metachronous metastatic group [12]. However, compared to synchronous mRCC, studies focused on the impact of targeted therapy on the prognosis and clinical outcomes of metachronous mRCC are limited. Accordingly, no specific prognostic model for metachronous mRCC has been introduced, whereas several prognostic risk groupings for whole mRCC have been demonstrated, including the Memorial Sloan–Kettering Cancer Center (MSKCC) criteria, the International mRCC Database Consortium (IMDC) risk criteria, and the UCLA Integrated Staging System [1, 13, 14].

Herein, we focused on the prognostic and predictive factors of time to treatment failure (TTF) and OS, respectively, as clinical parameters that are critical to targeted therapies in patients with metachronous mRCCs.

## RESULTS

In this study, retrospective reviews of 101 patients with metachronous RCC were conducted (Table 1). The mean age at diagnosis was 58.4±11.4 years and 73.3% were male. The histologic characteristics of the initial tumor included clear cell types (90.1%) and 68.3% were Fuhrman grade 3–4. Approximately, 10% exhibited sarcomatoid features and histologic necrosis. Patients with tumors with sarcomatoid features were placed in the Fuhrman grade 3–4 group. Half of the patients were stage T3, followed by T1 (29.7%) and T2 (19.8%). Single site metastasis was observed in 32.7% of the patients, with the lungs being the most common first metastasis site, followed by retroperitoneal space, bone, lymph node, and liver. Among 101 patients, death event occurred in 45 patients (44.6%). The median follow-up duration and time to metastasis were 37.0 [18.3, 59.4] and 13.2 [6.1, 34.1] months, respectively. The median TTF and OS were 19.2 [9.3, 40.2] and 23.6 [10.7. 38.5] months, respectively. Metastasectomy was performed in 40 patients (39.6%), and the most frequently performed surgery was lung wedge resection (35%, 14/40) followed by metastatic bone resection (22.5%, 9/40). Metastasectomy was both performed in single site metastasis and multiple site metastasis condition. Sunitinib was the most common first-line targeted agent (46.5%), followed by sorafenib (26.7%) and pazopanib (15.8%); however, no differences in OS or TTF were observed between the first line agents (p=0.706 and 0.872, respectively). The patients that underwent a metastasectomy received targeted therapy treatment for a median period of 16.3 [6.5, 35.6] months prior to the metastasectomy, and the treatments were resumed as an adjuvant setting following a mean period of 2.1 [0.5, 3.8] months after the metastasectomy.

**Table 1:** Baseline characteristics

Number of patients enrolled	101
Age at diagnosis of RCC, mean±SD (years)	58.4±11.4
Follow-up period after recurrence, median [IQR] (months)	37.0 [18.3, 59.4]
Gender, n (%)	
Male	74 (73.3)
Female	27 (26.7)
Histology, n (%)	
Clear cell	91 (90.1)
Non-clear cell	10 (9.9)
Fuhrman grade, n (%)	
G1	3 (3.0)
G2	26 (25.7)
G3	56 (55.4)
G4	13 (12.9)
Undefined	3 (3.0)
Sarcomatoid features, n (%)	10 (9.9)
Histological Necrosis, n (%)	9 (8.9)
Lymphovascular invasion, n (%)	23 (22.8)
Tumor stage	
pT1	30 (29.7)
pT2	20 (19.8)
pT3	51 (50.5)
Solitary site metastasis, n (%)	33 (32.7)
First site of metastasis, n (%)	
Retroperitoneal space	16 (15.8)
Lung	41 (40.6)
Liver	4 (4.0)
Bone	12 (11.9)
Brain	1 (1.0)
Lymph node	5 (5.0)
Others	22 (21.8)
Metastasectomy, n (%)	40 (39.6)
Time to metastasis from nephrectomy, median [IQR] (months)	13.2 [6.1, 34.1]
Time to initial treatment failure, median [IQR] (months)	19.2 [9.3, 40.2]
Time to death from metastasis, median [IQR] (months)	23.6 [10.7. 38.5]
Time to death from nephrectomy median [IQR] (months)	36.5 [19.2, 64.2]

**Figure 1:**
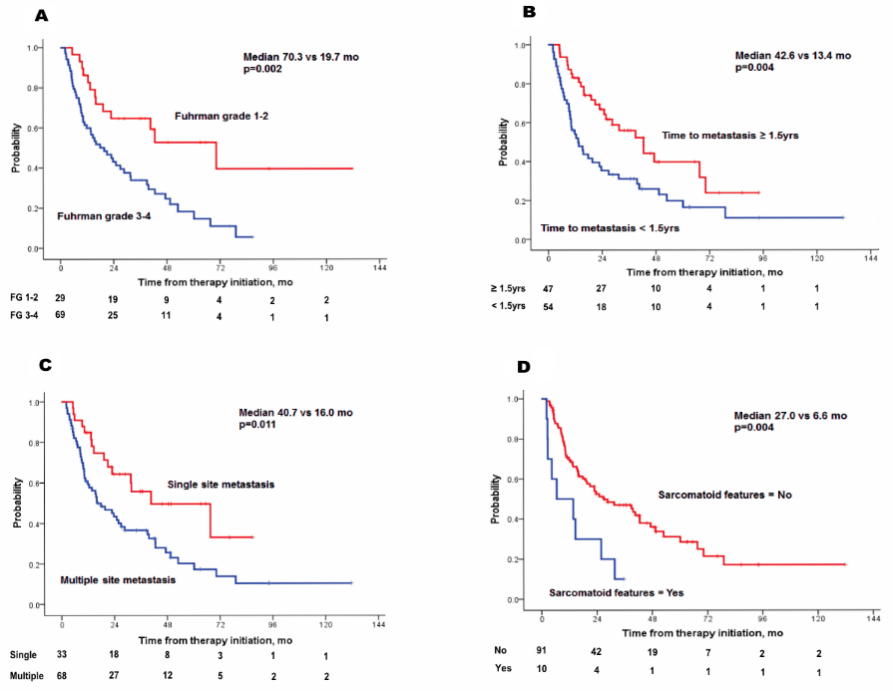
Kaplan-Meier plots of time to treatment failure by four risk factors **(A)** Fuhrman grade 3–4, **(B)** time to metastasis <1. 5 years, **(C)** multiple site metastasis, **(D)** sarcomatoid features.

**Figure 2:**
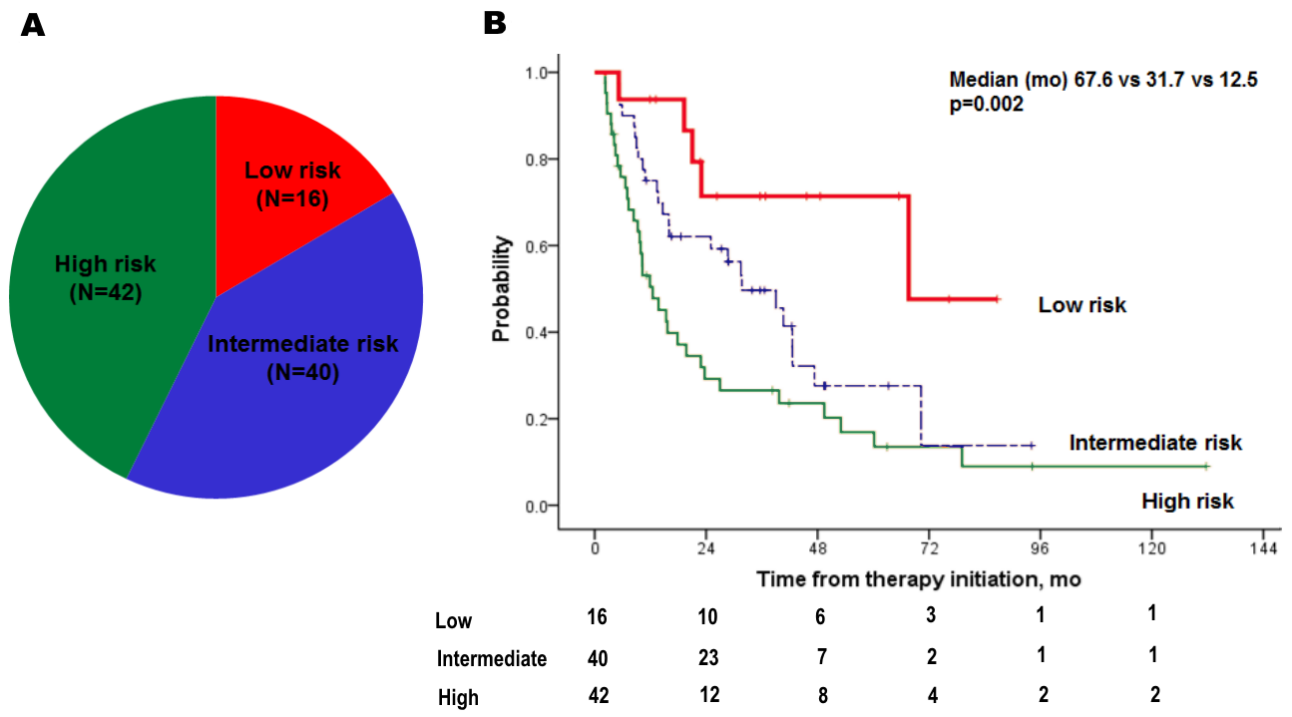
Distribution **(A)** and Kaplan-Meier plots **(B)** of time to treatment failure of three risk groups: low (risk factor=0), intermediate (risk factor=1), and high (risk factor ≥2).

Predictive factors for TTF were analyzed by Cox regression (Table 2). Sarcomatoid features [hazard ratio (HR), 4.208; p=0.001], higher Fuhrman grade (3–4; HR, 2.435; p=0.013), single metastatic site (HR, 0.455; p=0.030), and time to metastasis < 1.5 years (HR, 2.267; p=0.006) showed a significant impact on TTF (Table 2 and Figure 1) following the multivariate analyses. Using four independent factors, three risk groups were generated: low (risk factor=0; N=16, 16.3%), intermediate (risk factor=1; N=40, 40.8%) and high-risk (risk factor ≥2; N=42, 42.9%). A survival graph of each risk group was generated (Figure 2), which indicated significantly different TTF in the three risk groups (median TTF for low, intermediate, and high-risk groups: 67.6 vs 31.7 vs 12.5 months, respectively; p=0.002)

**Table 2:** Association of various factors with time to treatment failure in Cox proportional hazard regression analysis

	Univariate analysis			Multivariate analysis		
Variables (n)	HR	95% CI	P value	HR	95% CI	P value
Age: ≥60 years vs <60 years (53 vs 48)	1.157	0.711–1.884	0.558			
Sex: males vs females (74 vs 27)	0.815	0.461–1.442	0.482			
Size ≥4cm (87 vs 14)	1.329	0.632–1.329	0.453			
T stage			0.896			
pT1 (reference) (30)						
pT2 (20)	0.976	0.552–1.728	0.935			
pT3 (51)	1.135	0.612–2.105	0.687			
Histological type: clear cell vs others (91 vs 10)	0.904	0.388–2.105	0.815			
Sarcomatoid change (10)	2.745	1.340–5.623	0.006	4.208	1.736–10.196	0.001
Histologic necrosis (9)	1.157	0.528–2.537	0.716			
Fuhrman grade: Grade 3-4 vs 1-2 (69 vs 29)	2.554	1.383–4.716	0.003	2.435	1.203–4.928	0.013
Metastasectomy (40)	0.499	0.279–0.892	0.019	0.575	0.294–1.123	0.105
Metastatic sites: single vs multiple (33 vs 68)	0.482	0.271–0.858	0.013	0.455	0.223–0.928	0.030
Time to metastasis: <1.5yr vs ≥1.5yr (54 vs 47)	2.060	1.257–3.376	0.004	2.267	1.266–4.060	0.006
First metastasis: Retroperitoneal space (16)	1.133	0.576–2.227	0.717			
First metastasis: Lung (41)	0.830	0.508–1.354	0.455			
First metastasis: Liver (4)	1.929	0.697–5.343	0.206			
First metastasis: Bone (12)	1.124	0.556–2.273	0.745			
First metastasis: Lymph node (5)	1.065	0.333–3.405	0.915			
ASA: 3–4 vs 1–2 (26 vs 65)	0.907	0.503–1.637	0.746			
Hemoglobin (g/dL)	0.886	0.768–1.023	0.099			
LDH (IU/L)	1.005	0.996–1.015	0.258			
Corrected calcium (mg/dL)	2.468	1.164–5.231	0.018	1.969	0.946-4.099	0.070
eGFR (CKD-EPI) (ml/min/1.73m^2^)	0.992	0.975–1.008	0.319			

HR, hazard ratio; CI, confidence interval; ASA, American Society of Anesthesiologists; LDH, lactate dehydrogenase; eGFR, estimated glomerular filtration rate; CKD-EPI, Chronic Kidney Disease Epidemiology Collaboration.

**Figure 3:**
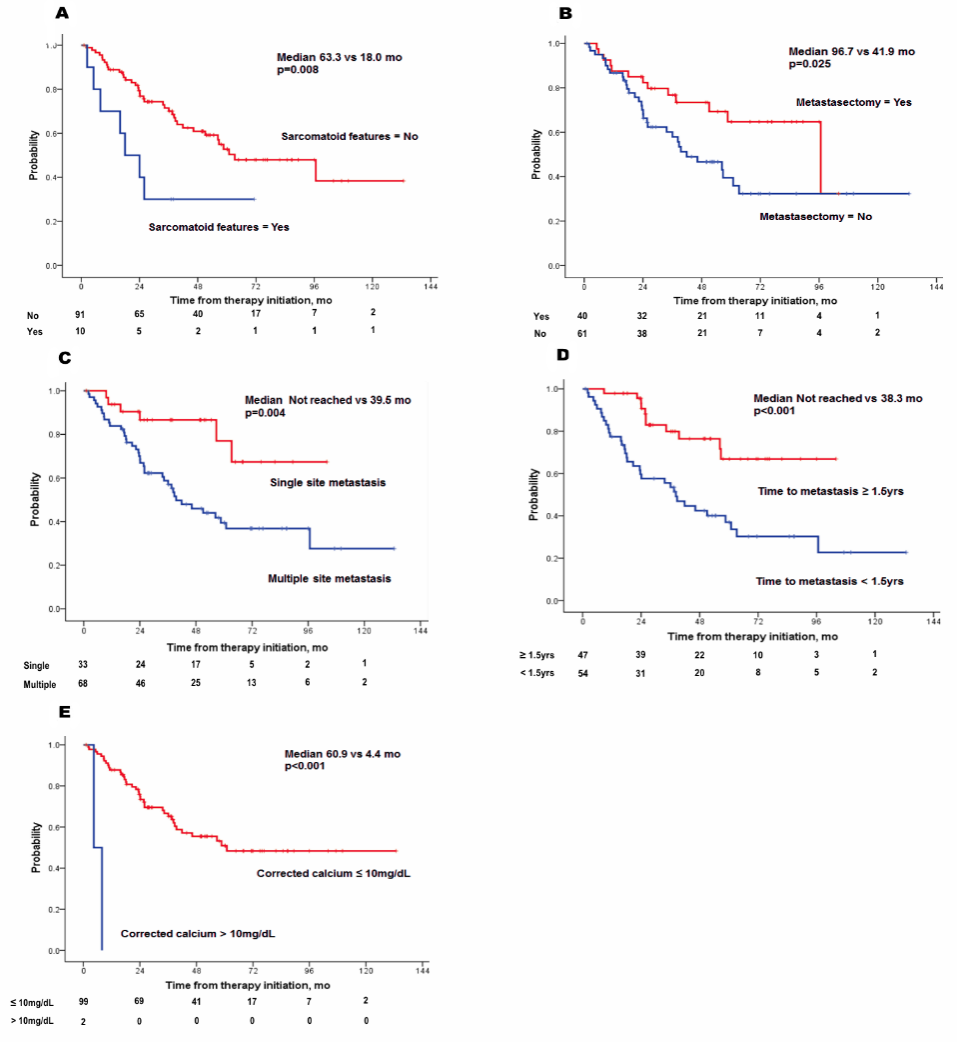
Kaplan-Meier plots of overall survival by five risk factors **(A)** sarcomatoid features, **(B)** absence of metastasectomy, **(C)** multiple site metastasis, **(D)** time to metastasis < 1. 5 years, **(E)** higher corrected calcium levels.

**Figure 4:**
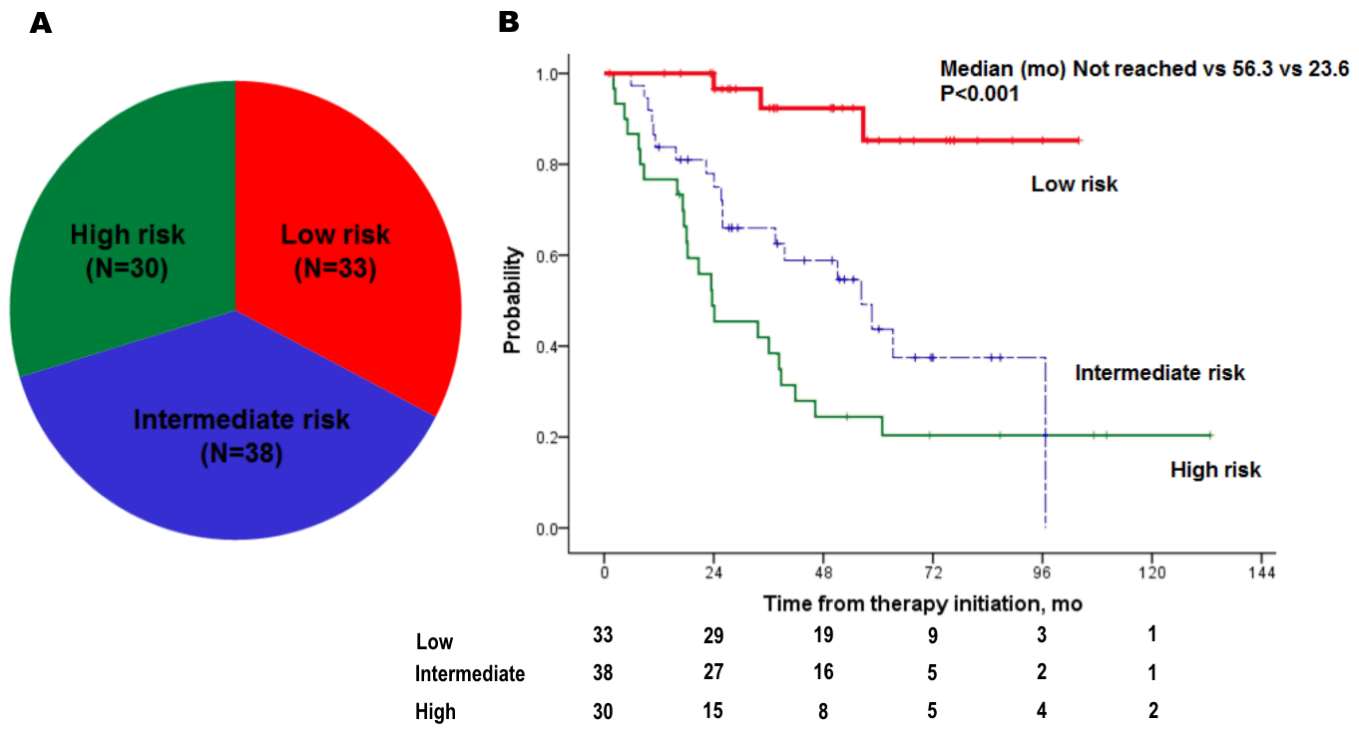
Distribution **(A)** and Kaplan-Meier plots **(B)** of overall survival of three risk groups: low (risk factor=0–1), intermediate (risk factor=2), and high (risk factor≥3).

Significant OS prediction factors were also analyzed by Cox regression (Table 3). Following multivariate analyses, sarcomatoid features (HR, 4.714; p=0.003), metastasectomy (HR, 0.437; p=0.045), single metastasis site (HR, 0.194; p=0.011), time to metastasis <1.5 years (HR, 3.053; p=0.011), and higher corrected calcium (for increase at every 1mg/dl) (HR, 5.607; p=0.001) were independent factors that affected OS (Figure 3). Using five independent factors, three risk groups were generated: low (risk factor=0–1), intermediate (risk factor=2), and high risk (risk factor=3–5). A survival graph of each risk group was generated (Figure 4), which demonstrated significant differences in OS between the groups (median OS in the low group was not reached, whereas OS for the intermediate and high risk groups were 58.6 and 23.6 months, respectively; p<0.001).

**Table 3:** Association of various factors with overall survival in Cox proportional hazard regression analysis

	Univariate analysis			Multivariate analysis		
Variables (n)	HR	95% CI	P value	HR	95% CI	P value
Age: ≥60 years vs <60 years (53 vs 48)	1.199	0.666–2.161	0.545			
Sex: males vs females (74 vs 27)	0.732	0.389–1.377	0.333			
Size ≥4 cm (87 vs 14)	1.653	0.591–4.620	0.338			
T stage			0.871			
pT1 (reference) (30)						
pT2 (20)	0.821	0.350–1.928	0.651			
pT3 (51)	0.999	0.505–1.975	0.998			
Histological type: clear cell vs others (91 vs 10)	0.734	0.260–2.072	0.559			
Sarcomatoid change (10)	2.865	1.267–6.480	0.011	4.714	1.711–12.987	0.003
Histologic necrosis (9)	1.714	0.724–4.059	0.220			
Fuhrman grade: Grade 3–4 vs 1–2 (69 vs 29)	1.999	0.988–4.044	0.054			
Metastasectomy (40)	0.484	0.253–0.925	0.028	0.437	0.194–0.983	0.045
Metastatic sites: single vs multiple (33 vs 68)	0.300	0.127–0.709	0.006	0.194	0.055–0.684	0.011
Time to metastasis: <1.5yr vs >1.5yr (54 vs 47)	3.313	1.675–6.553	0.001	3.053	1.291–7.221	0.011
First metastasis: Retroperitoneal space (16)	2.209	1.115–4.379	0.023	0.960	0.350–2.635	0.938
First metastasis: Lung (41)	0.345	0.170–0.698	0.003	0.830	0.345–1.998	0.677
First metastasis: Liver (4)	2.013	0.621–6.524	0.244			
First metastasis: Bone (12)	1.475	0.686–3.171	0.319			
First metastasis: Lymph node (5)	0.335	0.046–2.440	0.280			
ASA: 3–4 vs 1–2 (26 vs 65)	0.975	0.460–2.068	0.948			
Hemoglobin (g/dL)	0.798	0.677–0.940	0.007	0.958	0.786–1.168	0.673
LDH (IU/L)	1.004	0.994–1.015	0.420			
Corrected calcium (mg/dL)	6.433	2.499–16.562	<0.001	5.607	2.116–14.852	0.001
eGFR (CKD-EPI) (ml/min/1.73m^2^)	0.987	0.967–1.007	0.201			

HR, hazard ratio; CI, confidence interval; ASA, American Society of Anesthesiologists; LDH, lactate dehydrogenase; eGFR, estimated glomerular filtration rate; CKD-EPI, Chronic Kidney Disease Epidemiology Collaboration.

The predictive discrimination of the C-indexes calculated for our model and the IMDC and MSKCC models were compared. The Harrell’s C-index value of our model was 0.745 (95% CI = 0.671, 0.815), which was greater than the IMDC C-index value of 0.659 (95% CI = 0.568, 0.739) and the MSKCC C-index value of 0.680 (95% CI = 0.593, 0.755). The difference between the Harrell’s C-index of our model and the IMDC model was 0.087 (95% CI = 0.004, 0.172), whereas the difference between our model and the MSKCC model was 0.066 (95% CI = -0.017, 0.157). The AUC calculated by integrating over time was generally higher in our model compared to the IMDC and MSKCC models. When the death event served as the dependent factor, the area under the ROC curve (AUC) was significantly higher in our model compared to the other two models. The AUCs for our model and the IMDC model were 0.805 and 0.656, respectively (ours vs. IDMS, p=0.010), and the AUC for the MSKCC model was 0.659 (ours vs. MSKCC, p=0.010).

Univariate and multivariate logistic regression analysis was performed to sort the poor responders and those relatively resistant to targeted agents (Supplementary Table 1). The median duration of TKI treatment was 9.7 months (0.8–72.7). Within this period, the poor responder patients exhibited shorter OS than the good responders (median survival: not reached vs 24 months, p<0.001). Following univariate analyses, sarcomatoid features, time to metastasis <1.5 years, lower hemoglobin, and higher corrected calcium levels were predictive values of poor responders, whereas single metastasis site and first metastasis in the lung were predictive of good responders. The multivariate analyses demonstrated that only sarcomatoid features and single site metastasis independently predicted poor and good responders, respectively [Odds ratio (OR), 8.355; p=0.034 and OR, 0.218; p=0.016, respectively; Supplementary Figure 1].

**Figure 5:**
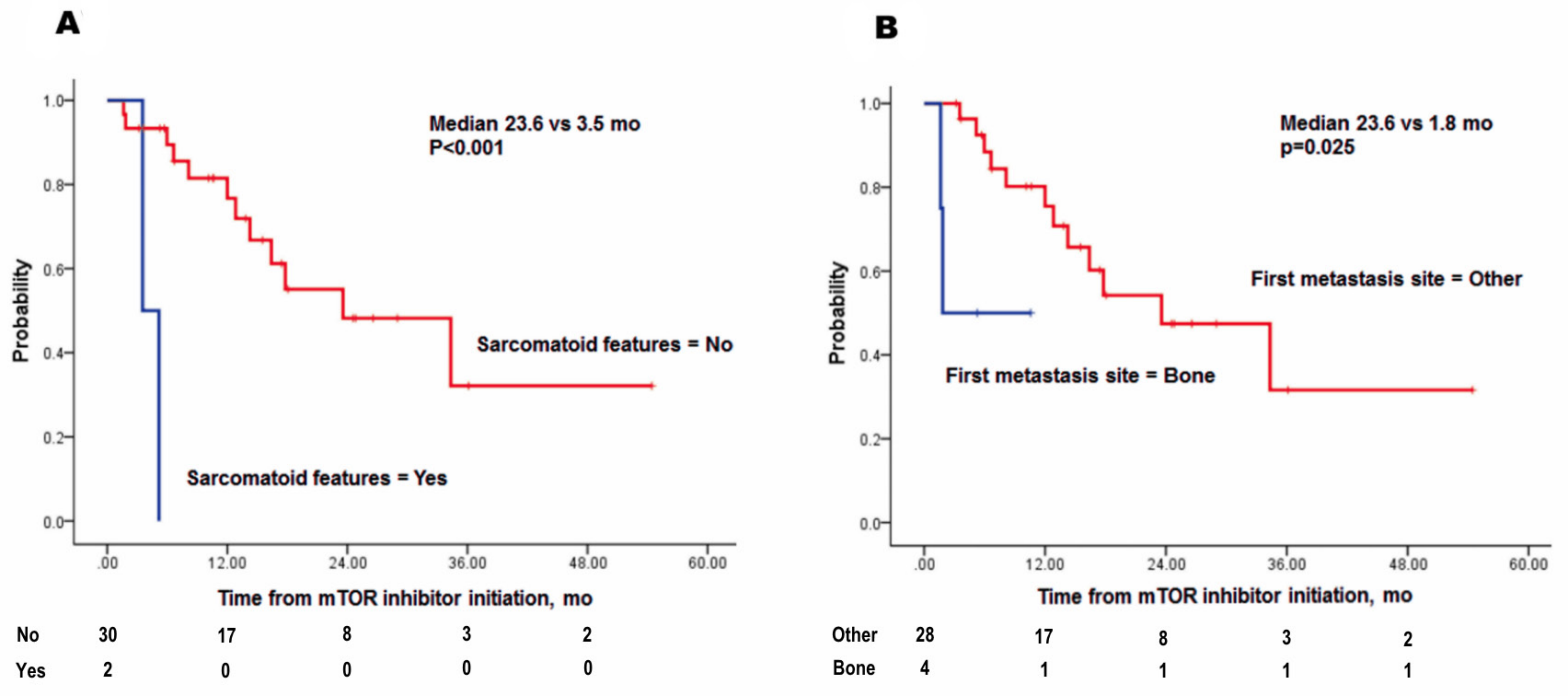
Kaplan-Meier plots of overall survival following initiation of a second line mTOR inhibitor by two risk factors **(A)** sarcomatoid features and **(B)** first site of metastasis to bone.

A sub-analysis was performed using Cox regression to predict OS in patients who received first-line TKI followed by a second-line mTOR inhibitor (Table 4). Total 32 patients were included in this group. 30 patients (93.8%) received everolimus, while remaining 2 patients (6.2%) received temsirolimus as second-line mTOR inhibitor. OS was defined as the time between the start of the second-line mTOR inhibitor to the date of death. The multivariate analysis revealed that sarcomatoid features (HR, 31.331; p=0.002) and first metastasis to bone (HR, 10.261; p=0.013) significantly influenced OS, whereas the duration of first-line TKI treatment was not associated with OS in the subjects (Figure 5).

**Table 4:** Association of various factors with overall survival in patients that received first-line TKI and second-line mTOR inhibitor using Cox proportional hazard regression analysis (n=32)

	Univariate analysis			Multivariate analysis		
Variables (n)	HR	95% CI	P value	HR	95% CI	P value
Age: ≥60 years vs <60 years (17 vs 15)	1.903	0.633–5.723	0.252			
Sex: males vs females (26 vs 6)	0.495	0.152–1.615	0.244			
Size ≥4cm (27 vs 5)	0.688	0.189–2.510	0.571			
T stage			0.977			
pT1 (reference) (10)						
pT2 (2)	0.00	0.00–0.00	0.990			
pT3 (20)	1.128	0.374–3.407	0.830			
Sarcomatoid change (2)	16.596	2.30–119.773	0.005	31.331	3.509–279.773	0.002
Histologic necrosis (3)	0.871	0.113–6.722	0.895			
Fuhrman’s grade: Grade 3–4 vs 1–2 (23 vs 9)	1.666	0.448–6.191	0.446			
Metastasectomy (13)	0.576	0.180–1.844	0.353			
Metastatic sites: solitary vs multiple (8 vs 24)	0.370	0.079–1.732	0.207			
Time to metastasis: <1.5yr vs >1.5yr (21 vs 11)	2.231	0.611–8.143	0.225			
First metastasis: Retroperitoneal space (3)	1.577	0.335–7.428	0.564			
First metastasis: Lung (16)	0.318	0.099–1.021	0.054			
First metastasis: Liver (3)	2.820	0.581–13.686	0.198			
First metastasis: Bone (4)	5.443	1.024–28.924	0.047	10.261	1.649–63.850	0.013
First metastasis: Lymph node (2)	0.680	0.087–5.295	0.712			
ASA: 3–4 vs 1–2 (4 vs 23)	0.485	0.062–3.767	0.489			
Initial TKI duration	0.998	0.995–1.000	0.073			
Hemoglobin (g/dL)	0.839	0.612–1.152	0.278			
LDH (IU/L)	0.931	0.748–1.160	0.525			
Corrected calcium (mg/dL)	3.720	0.880–15.732	0.074			
eGFR (CKD-EPI) (ml/min/1.73m^2^)	1.014	0.987–1.043	0.310			

HR, hazard ratio; CI, confidence interval; ASA, American Society of Anesthesiologists; TKI, tyrosine kinase inhibitor; LDH, lactate dehydrogenase; eGFR, estimated glomerular filtration rate; CKD-EPI, Chronic Kidney Disease Epidemiology Collaboration.

## DISCUSSION

Therapeutic options for mRCC have changed in recent years owing to the availability of targeted therapies [15], which have more than doubled the median OS for most mRCC patients [16]. Additionally, through lengthy and large cohort studies of various targeted agents, a variety of therapeutic agents with similar outcomes regarding OS and progression-free survival have been established [17].

We aimed to clarify the prognostic factors associated with metachronous mRCC in an attempt to prolong survival outcomes. Our study demonstrates interesting results regarding TTF and OS. Once the initial tumor was removed and residual tumor was absent, initial tumor size and T stage did not affect the prognosis in metachronous mRCC. Two prior studies have shown that size does not affect the prognosis of metachronous mRCC in small renal masses <4 cm [3, 18]. Nonetheless, size was a significant variable in the CORONA/SATURN-Project; however, the study primarily included patients treated with immune-based therapies from 1992–2010 [19]. As well, the CORONA/SATURN-Project results suggest that initial T stage is a significant factor for OS [19]. It has been established that higher T stage demonstrates worse OS in synchronous mRCC [20]. In the current study, sarcomatoid features demonstrated the highest HR in metachronous mRCC and affected both TTF and OS, which was in accord with previous studies [21]. Further, Fuhrman grade, a well-known prognostic factor for both TTF and OS in synchronous mRCC [22], independently affected TTF and exhibited borderline significance (p=0.054) with regards to OS. We additionally applied WHO/ISUP grading system and WHO/ISUP grade also significantly affected TTF (p=0.013), while having borderline significance at OS (p=0.065). Finally, the first metastasis site was not clinically relevant, but the number of metastatic sites was a significant factor.

No guidelines for managing patients with refractory mRCC or that are resistant to targeted agents have been establishedA 5-year survival rate of 30%–45% has been reported in patients with mRCC after metastasectomy and the complete resection of all metastases has been associated with a 2-fold decrease in the risk of death [23]. Further, several researchers have stressed the integration of medical therapy and surgical resection. Alt et al. insisted that the optimal management of patients with mRCC was a combination strategy [23]. Similarly, Santini et al. demonstrated that multimodal treatment could be a valid approach to overcoming tumor heterogeneity involved in TKI resistance [4]. Additionally, Karam et al. analyzed the clinical significance of metastasectomy after targeted therapy and concluded that approximately 50% had no recurrence at a median of 43 weeks after combining both modalities [7].

Nonetheless, more accurate and concrete recommendations are needed regarding metastasectomy and the implication of each metastasis site. For example, Alt et al. indicated that metastasectomy is only effective when a complete resection is performed [23]. Regarding the site of metastasis, a pulmonary metastasectomy is beneficial when it involves metachronous metastasis with a long disease-free interval and a relatively small metastasis burden [12]. Dabestani et al. reported that with the exception of brain and bone metastases, metastasectomy remains the most appropriate local treatment for most sites [24]. However, metastasectomy for other sites are still controversial.

Regarding the time to metastasis factor, Poel et al. showed that patients with an interval <2 years between primary tumor and metastasis have significantly shorter disease-specific survival intervals compared to those with intervals >2 years [25]. According to Webber et al., whose study included all mRCC with or without prior nephrectomy, the only baseline variable consistently related to OS, TTF, and response to first-line anti-VEGF TKI therapy was time from diagnosis to treatment >12 months. Brookman et al. reported that greater than 13,000 patients with initially localized RCC had metachronous metastasis [19]. In this large study, time to metastasis <12 months, initial tumor size, and stage were prognostic factors, which was not in accord with our results. However, the study included heterogeneous treatments involving both immunotherapy and TKI, and did not consider metastasectomy. In our study, metastasectomy was performed in 40 patients (39.6%) who showed prolonged OS. Metastasectomy could be one of the options for patients who demonstrate short OS despite TKI treatment. Regarding clinical efficacy of metastasectomy for different surgery site, further study should be followed in future study.

We generated a prognostic model for TTF and OS for metachronous mRCC by dividing mRCC into low, intermediate, and high-risk groups. In both risk group classifications, sarcomatoid features and time to metastasis <1.5 years were negative prognostic factors, whereas a single metastasis site was a positive prognostic factor. In clinical practice, these three factors should be carefully reviewed for each patient, and secondary modalities should be recommended along with the primary treatment.

Our model demonstrated statistically significant discrimination ability as a survival model compared to the IMDC and MSKCC models (Supplementary Figure 2). Because our model is specific to metachronous mRCC, we believe our model merits consideration for the application to patients with metachronous mRCC, which is correlated with relatively longer OS, compared to patients with synchronous mRCC, which is associated with more aggressive characteristics.

An mTOR inhibitor is one of the standard treatments for mRCC patients who fail initial TKI therapy [26, 27]. However, no prior studies have addressed prognostic factors for metachronous mRCC patients who received TKI treatment followed by an mTOR inhibitor. In this study, sarcomatoid features appeared the most powerful prognostic factor, exhibiting a clinical impact throughout the treatment period and a high HR. As previously demonstrated for synchronous mRCC, initial metastasis to bone was a predictor of poor outcome [28]. However, we should be very careful in interpreting and drawing meaningful conclusion out of this result, for the group size is small. There were 2 patients (6.3%) with sarcomatoid feature, and 4 patients (12.5%) with first metastasis to bone. For the sarcomatoid feature, each patient’s OS was 3.5 month and 5.2 months, while the median OS of non-sarcomatoid feature was 13.3 months [6.5, 23.8]. For the bone metastasis group, median OS was 3.6 months, while non-bone metastasis group showed median OS of 14.0 months [6.65, 24.3].

The limitations of our study include its retrospective nature and the relatively small number of patients. As well, there was no standardization of preoperative imaging or postoperative surveillance. However, at present no clear standardization approach has been established to guide such endeavors. And as pre-existing model of IMDC and MSKCC is validated in all tumor histological subtypes, we also included all the subtypes and validated our model overall. However for the distinct characteristics of each subtype, further study is needed for the additional validation with the sufficient number of patients. Finally, no standardization existed regarding the performance or extent of a lymph node dissection, which might have caused the actual incidence of patients with pN+ disease to be underestimated. Nonetheless, we believe our results are sufficient to suggest a trend as well as the feasibility of developing a specific prognostic model of targeted agents for metachronous mRCC.

In this era of targeted therapies, the initial tumor size and T stage did not affect TTF and OS in metachronous mRCC once nephrectomy was performed and residual tumor was absent (R0). Thus, our results indicate that the prognosis prediction models should not be applied to patients with metachronous mRCC that has developed from synchronous mRCC. Metastasectomy could prolong the survival time of metachronous mRCC patients. Those who cannot undergo metastasectomy who develop multiple metastasis in a relatively short time, have higher corrected calcium levels, and sarcomatoid features identified pathologically are more likely to benefit less from targeted agents regarding survival outcome. In addition, our results indicate that patients who have tumors with sarcomatoid features and bone metastasis are more likely to achieve fewer survival benefits from mTOR inhibitor therapy. Accordingly, new agents under development or in clinical trials, including novel VEGF inhibitors, immune checkpoint inhibitors, viral vaccines, or combination therapies, could be more beneficial to these high-risk patients.

## MATERIALS AND METHODS

### Good clinical practice protocols

The study was performed in accordance with applicable laws and regulations, good clinical practices, and ethical principles as described in the Declaration of Helsinki, and was approved by the Institutional Review Board.

### Patients

We retrospectively reviewed the medical charts of metachronous mRCC patients, defined as patients diagnosed with metastatic disease >3 months after the initial nephrectomy [29] with tumor relapse in the retroperitoneal space, lymph nodes, or other organs, treated at our institute between January 2005 and December 2015. Tumor relapse in the retroperitoneal space was defined as local recurrence, or recurrence within the retroperitoneal lymph nodes, the adrenal gland, or Gerota’s fascia. None of the patients received neoadjuvant or adjuvant treatment. All patients included in this study were surgically treated with radical nephrectomy or partial nephrectomy without evidence of residual tumor (R0). For the preoperative imaging study, we performed chest radiography and abdominopelvic computed tomography scans for all patients, as well as bone scans or brain imaging if there were any symptoms or clinical indications.

Patients with synchronous metastatic disease at presentation or diagnosis of metastatic disease <3 months after initial nephrectomy were excluded from the study, as well as patients who had received immune-based therapy as an initial systemic treatment, had secondary malignancies, underwent discontinuation of targeted agents due to toxicity with or without a doctor’s permission, and patients with a T4 pathological stage due to the potential for microscopic residual tumor after resection. Time to metastasis was defined as the time period from initial nephrectomy to the date of metachronous metastasis. The independent time variable was 1.5 years from nephrectomy because it was close to the inflection point of the receiver operating characteristic curve for both TTF and OS in this study (data not shown).

### Primary and secondary outcome measures

The primary outcome measures were TTF and OS, which were defined as the time between commencement of first-line targeted therapy and the date of progression and the time from the occurrence of disease metastasis to the date of death, respectively.

The secondary aim was to characterize treatment response to the targeted therapy agents. Targeted therapy treatment response was assessed nine months after commencement of targeted therapy because this was the median duration of initial targeted therapy in our study. Patients who survived and exhibited stable disease status following the first-line targeted therapy for ≥9 months were considered good responders, whereas those who were stable for <9 months were considered poor responders. Patients in complete remission before 9 months were categorized as good responders according to the Response Evaluation Criteria in Solid Tumors (RECIST v.1.1) [30].

Prognostic factors and OS were also analyzed in patients who received secondary mechanistic target of rapamycin (mTOR) inhibitor treatment following the administration of first-line tyrosine kinase inhibitors (TKI).

### Statistical analysis

Independent analyses were carried out to identify prognostic factors for investigator-assessed OS and TTF. Prognostic variables were based on a previously reported general review of pretreatment features [1, 13, 14]. Factors in the univariate analyses were assessed using the log-rank test. Statistically significant factors were then included in the multivariate Cox proportional hazard regression analysis to assess the influence of clinical and pathological parameters. Binary logistic regression analysis was performed to investigate the clinical factors that predicted the TKI response. The Harrell’s C-statistic was used to evaluate the performance of our model compared with the two previous prediction models (IMDC and MSKCC) and 95% confidence intervals (CI) of the resulting C-indexes were calculated by bootstrapping. One thousand bootstrap samples, which were generated by sampling the entire dataset of patients with replacement, were evaluated and 2-tailed 95% CIs were calculated. The 95% CIs of the pairwise differences between the C-indexes of the prognostic models were estimated using a similar approach. The area under the curve (AUC) was calculated by integrating over time to compare the performance trends or our model across survival time. The receiver-operating characteristic (ROC) curve was also used to analyze the performance of our risk model using the death event as the dependent variable. All statistical analyses were conducted using the “R” statistical software version 2.15.2.

## SUPPLEMENTARY MATERIALS FIGURES AND TABLE


