# Supplementary material for: Inherent characteristics of metachronous metastatic renal cell carcinoma in the era of targeted agents
Source: Oncotarget. 2017 Aug 12;8(45):78825–37. doi: 10.18632/oncotarget.20230 (PMC5668001; doi:10.18632/oncotarget.20230)
Supplement: Supplementary file 1 [file oncotarget-08-78825-s001.pdf]

Inherent characteristics of metachronous metastatic renal cell carcinoma in the Era of targeted agents

SUPPLEMENTARY MATERIALS

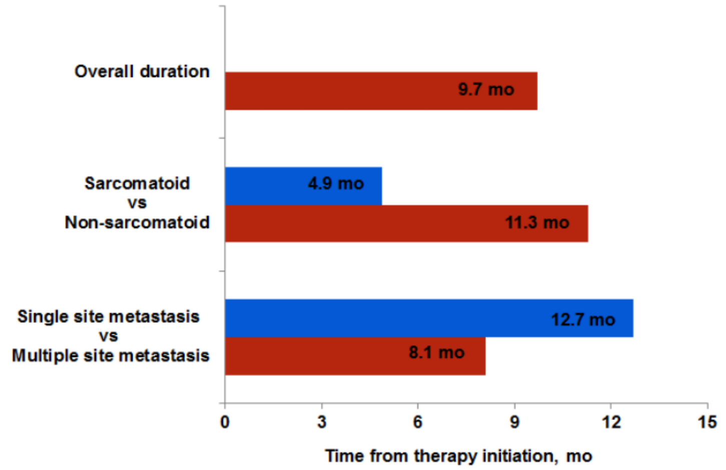

Supplementary Figure 1: Good and poor responders to initial targeted therapy agents and their median duration of use.

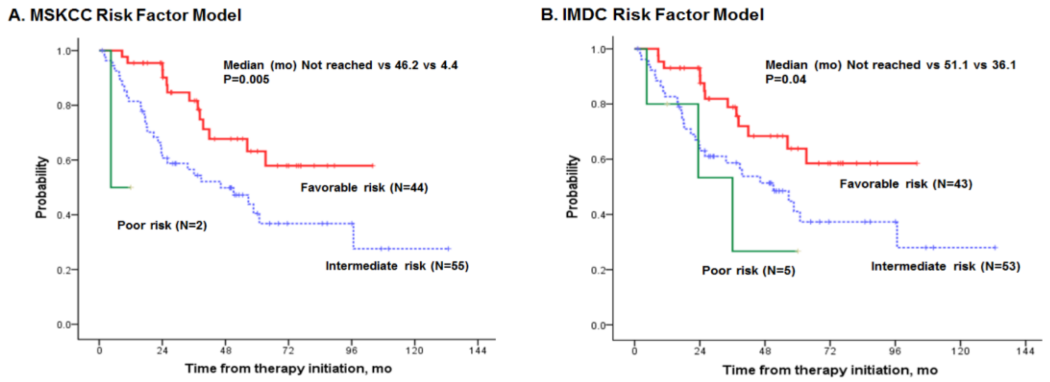

Supplementary Figure 2: Kaplan-Meier plots of overall survival determined by Memorial Sloan Kettering Cancer Center (MSKCC) **(A)** and International Metastatic Renal Cell Carcinoma Database Consortium (IMDC) **(B)**.

Supplementary Table 1: Analysis of factors associated with prediction of poor response (<9 months) in first-line TKI agents

	Univariate analysis			Multivariate analysis		
**Variables**	**OR**	**95% CI**	**P value**	**OR**	**95% CI**	**P value**
Age: ≥60 years vs <60 years	1.083	0.489–2.400	0.844			
Sex: males vs females	0.806	0.330–1.964	0.634			
Size ≥4cm	1.850	0.538–6.361	0.329			
T stage			0.819			
pT1 (reference)						
pT2	0.808	0.250–2.612	0.721			
pT3	1.138	0.455–2.846	0.782			
Histological type: clear cell vs others	0.424	0.112–1.611	0.208			
Sarcomatoid change	7.030	1.409–35.078	0.017	8.355	1.178–59.261	0.034
Histologic necrosis	0.711	0.167–3.019	0.643			
Fuhrman grade: Grade 3–4 vs 1–2	1.922	0.767–4.813	0.163			
Metastasectomy: done vs not done	0.607	0.242–1.520	0.286			
Metastatic sites: single vs multiple	0.269	0.103–0.703	0.007	0.218	0.063–0.752	0.016
Time to metastasis: <1.5yr vs ≥1.5yr	2.357	1.036–5.362	0.041	0.535	0.188–1.524	0.241
First metastasis: Retroperitoneal space	2.903	0.962–8.759	0.059			
First metastasis: Lung	0.302	0.126–0.723	0.007	0.506	0.162–1.583	0.241
First metastasis: Liver	1.487	0.201–11.006	0.698			
First metastasis: bone	3.394	0.948–12.146	0.060			
First metastasis: Lymph node	0.350	0.038–3.250	0.356			
ASA: 3–4 vs 1–2	0.745	0.289–1.920	0.542			
Hemoglobin (g/dL)	0.760	0.599–0.965	0.024	0.866	0.644–1.166	0.343
LDH (IU/L)	1.012	0.993–1.033	0.221			
Corrected calcium (mg/dL)	3.236	1.074–9.756	0.037	2.248	0.543–9.300	0.263
eGFR (CKD-EPI) (ml/min/1.73m^2)	0.987	0.963–1.011	0.291			

OR, odds ratio; CI, confidence interval; ASA, American Society of Anesthesiologists; LDH, lactate dehydrogenase; eGFR, estimated glomerular filtration rate; CKD-EPI, Chronic Kidney Disease Epidemiology Collaboration
